# Value change debt as a window of opportunity for transformative change: a case study on the mixed Indigenous food system of St. Paul Island, Alaska

**DOI:** 10.1007/s11625-025-01665-z

**Published:** 2025-03-21

**Authors:** Silja Zimmermann, Brian J. Dermody, Courtney Carothers, Valeria Di Fant, Lauren M. Divine, Kadyn Lestenkof-Zacharof, Veronica M. Padula, Bert Theunissen, Martin J. Wassen, Ine Dorresteijn

**Affiliations:** 1 Centre for Complex Systems Studies, Copernicus Institute of Sustainable Development, Utrecht University, Princetonlaan 8a, 3584 CB Utrecht, The Netherlands; 2 Copernicus Institute of Sustainable Development, Utrecht University, Princetonlaan 8a, 3584 CB Utrecht, The Netherlands; 3 College of Fisheries and Ocean Sciences, University of Alaska Fairbanks, 1007 W. 3 Ave, Suite 100, Anchorage, AK USA; 4 Deltares, Boussinesqweg 1, 2629 Delft, The Netherlands; 5 Department of Physical Geography, Utrecht University, Princetonlaan 8a, 3584 CB Utrecht, The Netherlands; 6 Aleut Community of St. Paul Island Ecosystem Conservation Office, St. Paul, Pribilof Islands, AK USA; 7 Descartes Centre for the History and Philosophy of Sciences and the Humanities, Utrecht University, Budapestlaan 6, 3584 CB Utrecht, The Netherlands

**Keywords:** Arctic Indigenous food systems, Social–ecological systems, Value pluralism, Leverage points, Cultural continuity, Interventions

## Abstract

Arctic Indigenous food systems are essential to the survival of local communities, but face significant challenges due to environmental, economic, and social pressures. The objective of this study is to elicit values of the mixed Indigenous food system on St. Paul Island, Alaska, and understand their potential as deep leverage points for transformative change in the context of their historical co-evolution with the local food system. To achieve this objective, we engage three generational groups within the Aleut Community of St. Paul Island to obtain local food system stories. From these stories, we (i) elicit historical events that are thought to have shaped the local food system, (ii) identify factors that influence the food system in its present and future states, and (iii) delineate intrinsic, instrumental, and relational food system values. Our findings show that most identified historical events are perceived to have undermined the Indigenous food system and that most factors identified to shape present and future food system states present barriers for community members to engage in traditional practices. Yet, despite this, values that relate to traditional Indigenous livelihoods remain central in the local value system. These results suggest a value change debt, i.e., a time lag between changes in peoples’ held values following changes in the system around them. We propose that this lag provides a window of opportunity to leverage transformative change. We argue that as long as traditional food system values persist, there is potential to reconfigure the food system in a way that embraces these values, enhancing the system's relevance to the community's way of life.

**Supplementary Information:**

The online version contains supplementary material available at 10.1007/s11625-025-01665-z.

## Introduction

Arctic Indigenous food systems are under threat from numerous challenges such as climate change (Berkes and Jolly 2001; Ford et al. 2009), declining subsistence species (Kenny et al. 2018a), escalating food prices (Kenny et al. 2018b), structural racism, criminalization of traditional ways of life (Carothers et al. 2021; Esquible et al. 2024; Stevens and Black 2019), and the erosion of Indigenous, traditional, and local knowledge (ILTK) (Soloway 2015). These threats undermine Indigenous sovereignty, traditional ways of life, and local food systems and lead to negative impacts on well-being and high levels of food insecurity for the communities that rely on them (Council of Canadian Academies 2014). Indigenous communities across the Arctic urgently need recognition and support to advance effective solutions to navigate the multifaceted challenges of the twenty-first century unfolding in their food systems and to safeguard their food sovereignty and security into the future (Nilsson and Evengard 2015; Huet et al. 2012).

Transforming a system into a more sustainable, equitable, and desirable state requires acknowledging the diverse and plural values held within that system (Belisle-Toler et al. 2021). Arctic Indigenous food systems hold value in, of, and for themselves (Gladun et al. 2021), provide diverse services to their communities (Markkula et al. 2019; Malinauskaite et al. 2019), and are repositories of cultural and social values relating to culture, traditions, sense of place, religion, and spirituality (Gould et al. 2019; Green et al. 2019; Sheremata 2018). Such intrinsic, instrumental, and relational values often coexist in people’s valuation of nature (Arias-Arévalo et al. 2017). Recognizing these diverse values holds potential for transformative change as they can be used to design solutions that are more responsive to community priorities and, hence, more likely to be implemented and supported by communities (Belisle-Toler et al. 2021). Values link to deep system levels, which are hard to target but have great potential for transformative change as how humans value the world fundamentally shapes their actions (Abson et al. 2017; Meadows 1999). Yet, systems can be structured in ways that prevent the expression of certain values, creating barriers for individuals and communities to act on what they hold most important (Guerrero and Wilson 2017). Addressing the system elements that inhibit value expression through targeted interventions that reconfigure the system to align with these values presents a critical opportunity for transformative change. Despite this potential, most assessments and interventions for Indigenous food systems to date address shallow system levels (parameters and feedbacks), while deep system levels remain underrepresented (Abson et al. 2017; Meadows 1999; Zimmermann et al. 2023; Donatuto et al. 2019). Understanding the role of values as potential levers for transformative change in Indigenous food systems hence presents an important knowledge gap.

Researchers have long acknowledged the importance of recognizing values in social–ecological systems (Arias-Arévalo et al. 2018). Diverse value articulation methods include monetary valuation of ecosystems and biodiversity (Sukhdev 2010) as well as qualitative assessments of community values (Reid et al. 2014; Berliner et al. 2011). As values co-evolve with the systems they are embedded in, another promising way to understand and analyze values is to view them in the historical context in which they arose (Horcea-Milcu et al. 2017). People ascribe importance to past events and experiences through valuation processes. They then reinforce these values through their actions, which in turn shapes their behavior in the present and future (Flood et al. 2021; Schultz et al. 2005). Only with this understanding is transformation possible in the present action space, where processes of valuing are linked to modes of action (Flood et al. 2021). This process leads to the establishment of robust values that underpin communities and can potentially be deep leverage points for transformative change if communities are enabled to act upon their values and live fully according to them (Abson et al. 2017; Meadows 1999). An effective means to uncover community values and the processes through which they have evolved is through stories.

Indigenous storytelling is a form of moral testimony to help communities face challenges and pursue opportunities in the face of change (Anthony 2013; Scroggie 2009). For Indigenous peoples, storytelling is a traditional way of intergenerational knowledge transmission and an important method of teaching cultural beliefs and values (Datta 2017; Archibald 2008). Most research to date has focused on the role of Elders (Larsen and Fondahl 2015; Karlsdóttir and Jungsberg 2015), who transmit intergenerational memory and knowledge through stories, ensuring the continuation of Indigenous epistemic traditions (Fernández-Llamazares and Cabeza 2018). While we agree that it is important to recognize the key role of Elders, there is a need for the involvement of all generations (Ford et al. 2012), as non-Elder adults and youth are underrepresented in current research (Rana et al. 2020), and interactions between different generations are declining, leading to less intergenerational knowledge transfer (Fernández-Llamazares and Cabeza 2018; Singh et al. 2010). Including non-Elder adults and youth besides Elders is particularly relevant in this study, as we are interested in historical events and factors that influence the local food system in its present and future states. Non-Elder adults and youth link past values as transferred to them via storytelling by Elders and the present through experiential learning and shape the future through their actions as they age.

In this study, we examine the potential role of values as deep leverage points for transformative change in the context of their historical co-evolution with an Indigenous food system. Our case study is focused on St. Paul Island, Alaska, and its mixed Indigenous food system in the Bering Sea (Zimmermann et al. 2024). This focus is borne out of a larger project focused on understanding how to leverage a transformation in the St. Paul Island food system to increase local food security (Appendix A) and because it presents an important case of an Indigenous mixed food system, where local Indigenous values mix with Western values. We formulate three sub-objectives to achieve the overarching aim of this study: (i) to understand which past experiences and historical events youth, non-Elder adults, and Elders in the St. Paul Island community narrate in local food system stories; (ii) to explore which factors youth, non-Elder adults, and Elders in the St. Paul Island community identify to influence the present and future food system; and (iii) to elicit the values youth, non-Elder adults, and Elders hold in the St. Paul Island food system. We work towards these objectives by employing a twofold approach that includes informal conversations and narrative interviews in storytelling sessions with three generations in the Aleut Community of St. Paul Island (ACSPI), the federally recognized tribe on St. Paul Island.

## Theoretical framework

**Fig. 1:**
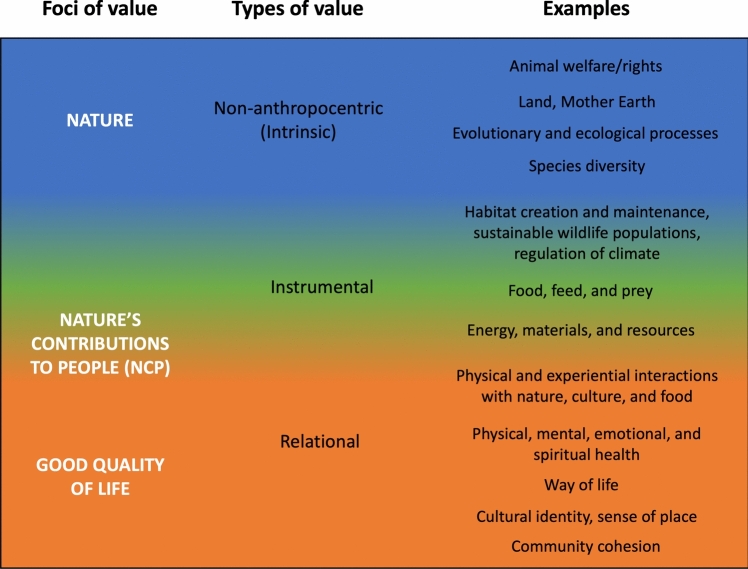
Diverse values related to nature, nature’s contributions to people (NCP), and a good quality of life. The color grading indicates that instrumental and relational values can be ascribed to the value of NCP and highlights that NCP is intertwined with nature and a good quality of life. The figure was adapted from Pascual et al. (2017)

Values are extremely diverse across different cultures and worldviews, and so are their scientific conceptualizations (Díaz et al. 2015; Pascual et al. 2017; Gould et al. 2019). The IPBES (Intergovernmental Science-Policy Platform on Biodiversity and Ecosystem Services) Conceptual Framework (hereafter: IPBES CF) provides a comprehensive framework to navigate such value diversity. It provides a shared language across multiple valuations, aiming to inform policy and practice and contribute to transformations (Díaz et al. 2015). The IPBES CF distinguishes between three foci of value in the interaction between the human and the non-human worlds, namely: nature (non-anthropocentric values: *intrinsic*); nature's contributions to people (anthropocentric values: *instrumental*); and good quality of life (anthropocentric values: *relational*) (Fig. 1).

Non-anthropocentric or intrinsic values (nature) refer to the value of nature, ecosystems, or life as ends in themselves and often provide the basis for moral duties (Arias-Arévalo et al. 2017, 2018). The term nature's contributions to people (NCP) resonates with the original use of the term ecosystem services, but goes further in that it explicitly embraces valuations associated with alternative, non-Western, and Indigenous knowledge systems. NCP refers to the benefits people draw from nature, social–ecological systems, or entity functions and has traditionally received the most attention in the ecosystem services literature using monetary valuation methods (Abson et al. 2014; Gómez-Baggethun and Martín-López 2015; Díaz et al. 2015). While the term is mostly associated with instrumental values, it can also include relational values and is further intertwined with all three IPBES CF foci. Good quality of life concerns the contribution of nature and ecosystem processes to a fulfilled human life (IPBES 2015). This third focus, thus, mostly contains relational values, embracing all kinds of relationships between people and nature, both on an individual and a collective level (Chan et al. 2018). Relational values recently attracted particular attention as intrinsic and instrumental values alone have been found insufficient to capture valuation processes fully (Chan et al. 2016). Especially in Indigenous contexts, relational values have been identified as an indispensable categorization (Chan et al. 2018; Pascual et al. 2017; Sheremata 2018; Gould et al. 2019).

The IPBES CF categorization of values emphasizes that values are fluid and sometimes cannot be placed into one specific category. Value pluralism recognizes the coexistence of multiple values that can sometimes conflict with or complement each other (Arias-Arévalo et al. 2018). Exploring value pluralism has gained momentum in social–ecological systems research in recent years (Chan et al. 2016; Kenter et al. 2019; Jacobs et al. 2016; Arias-Arévalo et al. 2018). In Indigenous contexts, acknowledging value pluralism is crucial as it respects the diverse range of values Indigenous communities attach to their environment and resources (Todd 2015).

Leverage points (LP) provide an analytical framework for identifying places within complex systems where targeted interventions can lead to transformative change. Meadows (1999) proposed a set of 12 places to intervene in a system and leverage system-wide change. These places, or LP, are organized in increasing order of effectiveness, with shallow LP referring to interventions that are relatively easy to implement but have a limited potential to lead to transformative change, and deep LP referring to interventions that are typically considered more difficult to act upon but have greater transformative potential (Meadows 1999; Abson et al. 2017). Abson et al. (2017) extended this work in the context of sustainability by matching the 12 LP with four system levels (parameters, feedbacks, design, intent) (Abson et al. 2017). Shallow system levels (parameters and feedback) conform with shallow LP and deep system levels (design and intent) with deep LP. Values are potentially deep LP for transformative change, because how humans value the world fundamentally shapes their actions (Abson et al. 2017; Schultz et al. 2005). Deep LP, such as the values, goals, and paradigms from which a system arises, are considered particularly powerful for transformative change, but also difficult to change (Meadows 1999). Values emerge over time (Horcea-Milcu et al. 2017), and people assign value through the active processes of remembering the past, engaging and interacting with a given system in the present, and restore value for the future through different activities (Flood et al. 2021). Feeding back into practices, institutions, and attitudes, these values maintain or shift the system from within (Horcea-Milcu et al. 2017). Due to this historical co-evolution, values do not stand alone but must be viewed in the historical context in which they arose to understand and analyze them. One way to elicit historical events and their valuation by people is through stories.

Stories are a basic element of human communication and can be found in all societies, cultures, and parts of life. People tell stories to make sense of their lives and create a shared understanding of past and current experiences (Groleau et al. 2006). Stories play a particularly large role in Indigenous communities, where they are an important way of transmitting knowledge from one generation to another (Datta 2017; Scroggie 2009; Cajete 1994). Indigenous communities have historically prioritized oral transmission of history over rendering it in written text, and today, storytelling remains of significant cultural, religious, and traditional importance for many communities (Datta 2017; Cidro 2012; Barnhardt and Kawagley 2005). Indigenous stories often hold a holistic and interconnected approach to knowledge that promotes collaboration, reciprocity, spirituality, and humility (Fernández-Llamazares and Cabeza 2018). They have previously been described as normative moral testimonies that represent the reservoir of collective wisdom of a community (Anthony 2013). Indigenous stories may examine past and current experiences to foster local understandings and often revolve around relationships between humans, food sources, and environmental ethics (Iseke 2013). Hence, Indigenous stories document both learning and reflection processes about social–ecological interconnections and can help us elicit events that impact or have impacted the food system and their moral integration (valuation) by community members (Archibald 2008).

A useful research technique to inspire stories is narrative interviewing (NI). NI is a qualitative research method that takes an unstructured approach to place the interviewee at the center of the interview and encourages them to tell a story or stories about a significant event in their life and social context (Jovchelovitch and Bauer 2000). NI has been particularly useful in projects that combine life stories with socio-historical contexts to understand the beliefs and values that motivate the actions of interviewees (Muylaert et al. 2014). NI has developed as a critique of the strictly guided style of most other interview techniques and aims to provoke stories close to the interviewees’ perspectives (Groleau et al. 2006). NI has proven suitable for encouraging storytelling and is, therefore, well compatible with Indigenous storytelling (Jovchelovitch and Bauer 2000). Indigenous methodologies, such as storytelling, have served as valuable research methods while remaining culturally meaningful to the communities involved (Kovach 2021; Cidro 2012). Utilizing such methods facilitates the preservation of Indigenous voices, builds resilience, and strengthens community empowerment (Iseke 2013; Dei 2011). Indigenous storytelling as a research method has been found to promote local participation, mitigate power imbalances between researchers and communities, and produce results with more tangible impacts in real life (Kovach 2021; Fernández-Llamazares and Cabeza 2018; Datta 2017). In this study, we, therefore, organize storytelling sessions with ACSPI members living on St. Paul Island, in which we conduct NI to extract local food system stories and identify events and factors that impact or have impacted the local food system and their valuation by community members. The IPBES CF provides the guiding framework for analyzing the conducted interviews and navigating value diversity (Kenter et al. 2019). We consider the IPBES CF particularly suitable for this study, as it incorporates value categories that resonate deeply with Indigenous worldviews and allows us to differentiate between diverse intrinsic, instrumental, and relational values.

## Methods

### Study area

**Fig. 2:**
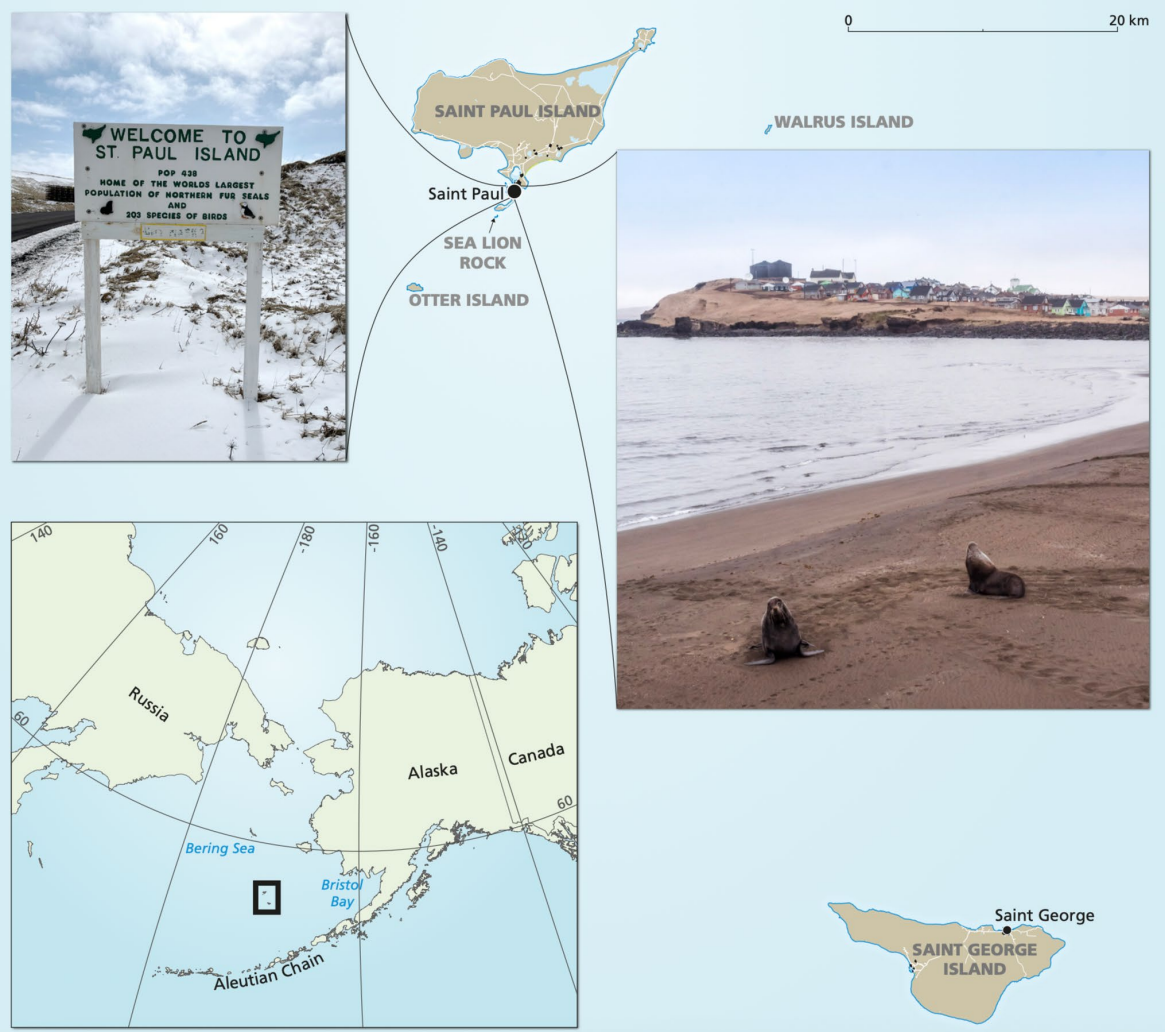
The five Pribilof Islands: St. Paul Island, St. George Island, Otter Island, Walrus Island, and Sea Lion Rock. The photos at the top left and mid-right of the map were taken by SZ in St. Paul on St. Paul Island. The overview map at the bottom left shows the location of the five Pribilof Islands and their wider surroundings in the southeastern Bering Sea

St. Paul Island is the largest of the five Pribilof Islands, located in the southeastern Bering Sea about ~ 400 km (250 mi) north of the Aleutian Chain and ~ 480 km (300 mi) west of the Alaska mainland. St. Paul has one small village with an estimated 379 residents, with > 85% identifying as Unangax̂ or other Alaska Native (World Population Review 2023). St. Paul’s population peaked in the 1990s and has been declining since, with a 6% decline since 2017 (TGSPI 2023). Today, an estimated 29% of the ACSPI are youth (0–18 years), 45% non-Elder adults (19–55 years), and 26% Elders (56–99 years) (TGSPI 2023). St. Paul has one school with 48 students enrolled for grades K-12 in 2023 (TGSPI 2023). Like many Indigenous communities, the Aleut Community of St. Paul Island (ACSPI) relies on a mixed economy, combining a cash-based wage economy with subsistence harvesting and sharing resources (Kruse et al. 2008). The St. Paul Island food system combines the Aleut Community Store and the subsistence harvest of laaqudax̂ (northern fur seal, *Callorhinus ursinus*), qawax̂ (Steller sea lion, *Eumatopias jubatus*), itayax̂ (domesticated reindeer, *Rangifer tarandus tarandus*), chagix̂ (Pacific halibut, *Hippoglogssus stenolepi*s), qimgiitan (multiple crab species), intertidal foods such as sea urchins, berries, and sometimes seabirds and eggs. St. Paul residents mostly obtain their food from the local store (*n* = 32; 100%), from traditional subsistence sources (*n* = 23; 72%), online orders (*n* = 20; 63%), and stores in Anchorage or other communities outside of the region (*n* = 16; 59%) (APIA 2024). The report also showed that 56% of the surveyed St. Paul residents often or sometimes worry that their food will run out before they have money to buy more and that 44% feel no or only a low level of control over getting the foods they want in St. Paul (APIA 2024) (Fig. 2).

## Research co-design

**Fig. 3:**
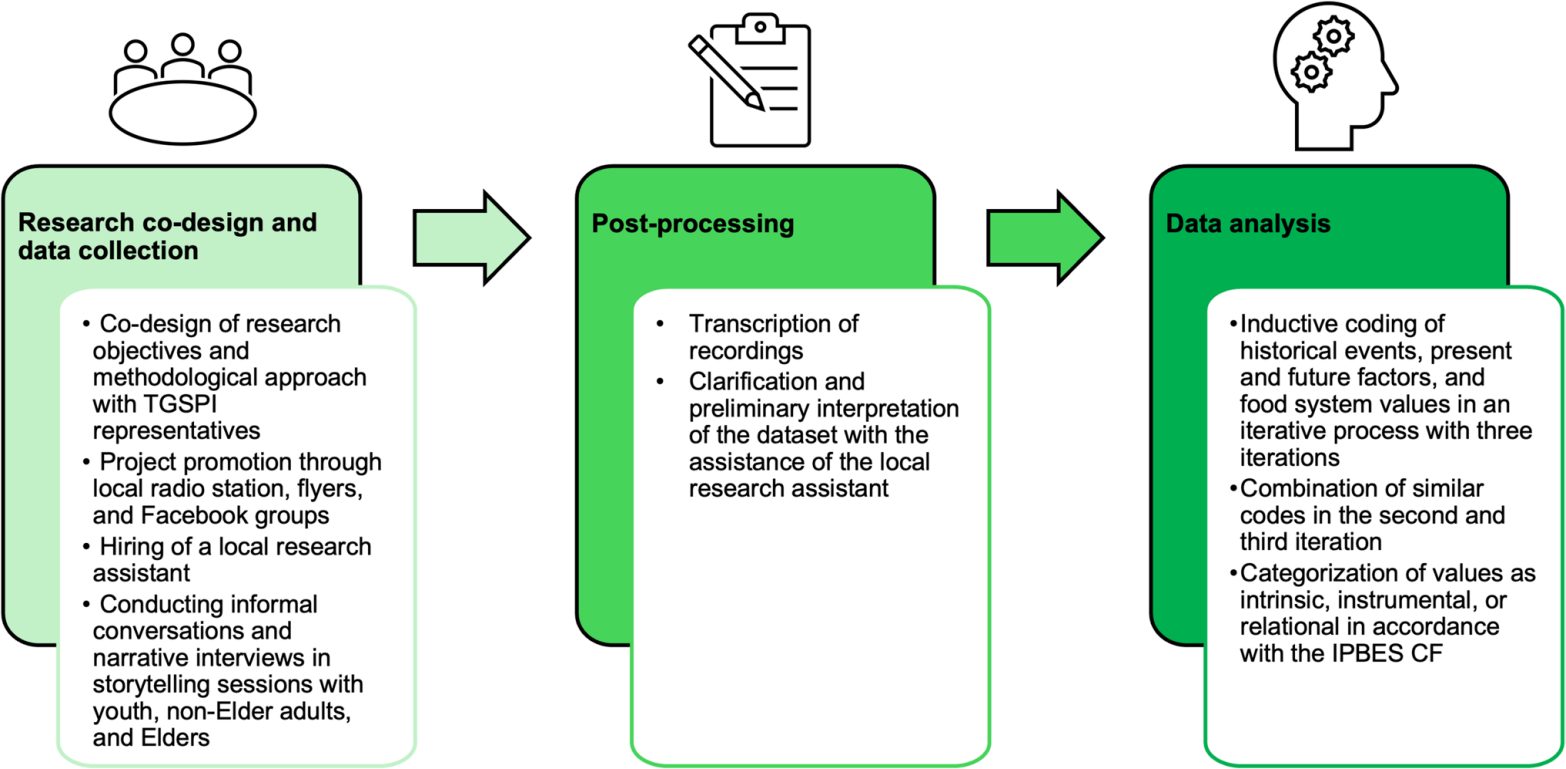
The workflow from research co-design and data collection to post-processing interview data and data analysis

The research objectives and methodological approach presented in this paper were co-designed by the authors and representatives of the Tribal Government of St. Paul Island (TGSPI) (Fig. 3) and take place in the context of a wider research project aimed at leveraging a transformation in the St. Paul Island food system to increase local food security. The author team has diverse backgrounds and levels of familiarity with the island (Appendix B). It was clear from conversations with TGSPI representatives that ACSPI members experience high levels of food insecurity and that narrative plays an important role in the community. We, hence, developed a narrative-based approach to elicit historical events and factors that influence the local food system in its present and future states, as well as the values ACSPI members hold in the St. Paul Island food system from stories. Meetings with TGSPI representatives to co-design the methodological approach occurred between February 2021 and January 2022. The findings presented in this paper will be built upon in succeeding research to co-design actionable pathways along which the ACSPI and their TGSPI can implement specific interventions to initiate a transformation toward a diverse and food-secure mixed food system on St. Paul Island (Appendix A).

### Data collection

Data were collected from March to May 2022 by SZ and KLZ through (1) informal conversations and (2) narrative interviews in storytelling sessions with ACSPI youth, non-Elder adults, and Elders. The interviews were conducted by SZ, a white, non-Indigenous female from Germany who is the PhD candidate of this project, with the support of KLZ, an Unangax̂ woman who has grown up on Tanax̂ Amix̂ (St. Paul Island) her whole life. Interviewees self-defined as youth, non-Elder adults, and Elders. Upon arrival on St. Paul Island in March 2022, SZ first aimed to become familiar with the local conditions by engaging in informal conversations with several ACSPI members. SZ participated in numerous food-related activities, including reindeer hunting, halibut fishing, community events, and grocery shopping, and engaged in informal conversations throughout these activities. The conversations were not recorded, but SZ carried a field diary to take notes after the conversations occurred. Based on the understanding gained from the informal encounters, SZ formulated two main questions to obtain stories from the interviewees during narrative interviews:

- Can you tell me a story about something that has to do with food? (*individual*)
- What are some of the most important stories about food that you remember being told on the island? (*collective*)

SZ hired KLZ as a local research assistant and promoted the project through the local radio station, flyers, and posting to relevant Facebook groups. Interested ACSPI members were invited to contact SZ to schedule a storytelling session. Additionally, VD, LD, and KLZ identified ACSPI members who they thought were relevant to interview owing to their community role and status. These individuals were approached personally by KLZ and SZ and invited for an interview. Interviewees were invited to choose the setting of each session to create a safe space for them to share their stories. All interviewees chose to hold their sessions at the local school or at their workplace. Each session lasted approximately 60 min, and all interviewees were compensated for their time. All sessions were conducted in English and audio-recorded (Fig. 3).

NI starts with the preparation of the interview. The interview itself has four phases: (i) initiation, (ii) main narration, (iii) questioning, and (iv) concluding talk (Jovchelovitch and Bauer 2000). However, NI's open and undirected format can make it difficult for interviewees to fully engage with the interview (Jovchelovitch and Bauer 2000). To tackle such issues, NI can be combined with semi-structured interview techniques, where the researcher has a topic guide at hand (Anderson and Kirkpatrick 2016). In this study, we followed the four phases of NI, but had a topic guide to fall back on and combined NI and semi-structured interview methods when needed (e.g., if the interview initiation did not inspire the interviewee to tell a story; Appendix C).

We started each session by informing the interviewees about the study’s context and our research aims, namely to hear stories about the food system, learn about events and factors that shaped or may shape the food system, and understand the values that are held in the food system. Interviewees were asked to provide free, informed consent to participate in the study. We then asked a set of general questions to allow the interviewees to introduce and position themselves in the community and the local food system (phase i). Next, we asked the two main questions: one aiming to obtain individual stories and one aiming to obtain collective stories. During the interviewees’ main narration, SZ and KLZ did not interrupt the interviewees but actively listened until the story’s end (ii). Following this, SZ and KLZ asked follow-up questions about the stories shared by the interviewees and additional questions inspired by the topic guide using the interviewees’ language to ensure the research aims could be met (iii). Lastly, SZ and KLZ conducted a concluding talk with the interviewees to gather further contextual information (iv). A detailed interview guide is given in Appendix C.

### Data post-processing and analysis

The session recordings were transcribed and coded by SZ using qualitative thematic analysis of the written transcripts with the software NVivo 14. Before processing with further analysis, SZ and the local research assistant (KLZ) discussed each interview’s content and KLZ provided background information on local contexts that were not apparent or unclear to SZ. Thereafter, SZ used an inductive bottom-up approach from the session transcripts to code for historical events that shaped the food system in the past, present factors that determine the food system today, future factors that are expected to influence the food system in the future, and food system values. The historical events, present and future factors, and values were identified in an iterative process with three iterations. Similar codes were combined by SZ in the second and third iterations (Fig. 3). Values were further categorized as intrinsic, instrumental, or relational. For each interview, the final codes were marked to indicate if they were applicable (0 = no, 1 = yes). Frequencies of the codes per interview were not recorded. The insights gained from the informal encounters were further used to complement our understanding of the food system and the values held by ACSPI members.

## Results

### General description of results

We conducted 19 narrative interviews, of which six interviewees were female and 13 were male. By generation category, five were with youth, nine with non-Elder adults, and five with Elders. Youth interviewees consisted of four boys and one girl, all enrolled as students at the local school. Non-Elder adults comprised six men and three women. Four of the non-Elder adult men were locally employed in St. Paul and two were unemployed. Elder interviewees included three men and two women, of whom two males were retired, while the others were locally employed in St. Paul. We identified 12 historical events that, according to the interviewees, have shaped the St. Paul Island food system in the past (objective (i)). Second, we identified 20 factors that, according to the interviewees, currently shape and are expected to shape the local food system in the future (objective (ii)). Third, we identified six intrinsic, 14 instrumental, and eight relational food system values across all interviews (objective (iii)). For all three objectives, non-Elder adults and Elders brought forward marginally more historical events, factors, and values than youth, but since the results were similar across all generations, we abstain from further elaborating on intergenerational differences in the results section (Table 1; Appendix D).

**Table 1:** Intrinsic, instrumental, and relational food system values, as identified from the stories and narrative interviews with youth, non-elder adults, and Elders

	Youth (*n* = 5)	Adults (*n* = 9)	Elders (*n* = 5)	Total count	Quotes
Intrinsic value					
Historical awareness	4	7	5	16	*“You know, we were brought here by the Russians and we were technically enslaved, we were forced to kill seals and it was only for their fur. My assumption is that over time, my people just learned to eat it because […] that’s all they were given, the only options.”*—Adult #3
Appreciation and respect for culture	4	8	3	15	*“I think it's good that […] is trying to keep the language and culture alive. I think that's really good.”*—Adult #2
Environmental awareness	4	6	5	15	*“Less crab, less halibut, less seals. […] It's the environment, the warmer oceans. I'm really glad the ice pack came. I'm really hoping it helps our ocean this year.”—*Adult #3
Appreciation and respect for nature and the environment	2	5	4	11	*“We have to respect our animals, our land and water.”*—Adult #7
Work ethic and dedication	2	5	3	10	*“I like helping around and doing stuff. […] Helping around just with anything, anybody needs, like bagging [at the community seal harvest] or any other thing.”*—Youth #4
Authenticity in food sources	2	3	3	8	*“With subsistence, […] I like that a lot better [than grocery store foods] because I know what it is and what's put in it.”*—Youth #4
Instrumental value					
Good taste	4	7	5	16	*“Oh my God. […] It's good, good food. I love my favorite foods. It tastes good.”*—Elder #1
Community support and sharing	3	7	5	15	*“Any subsistence foods were pretty much shared by the community with my family, with my dad. […] My dad got some great dishes of food from some wonderful cooks.”*—Elder #4
Fun and joy	2	7	4	13	*“I enjoy being on the water, so it's never work for me. You know, it's all joy.”*—Adult #6
Autonomy and independence	3	3	5	11	*“I want to make sure that my girls know how to provide for themselves. That's what drives me to hunt. The main reason I do hunt now is just for survival.”*—Adult #4
Health and well-being	2	5	3	10	“*What they have there keeps the very few of us, Unangan, that are living on this planet going in a healthy way, versus becoming diabetic, obese, having other health issues.”*—Elder #4
Environmental sustainability	0	6	4	10	*“And the ice coming down is good for the water temperatures and all the copepods and the small animals that feed the fish, birds, and stuff like that. So maybe we will see more birds surviving. And if we get more fish, then more seals will survive and stuff like that.”*—Elder #2
Economic sustainability	0	7	3	10	*“Halibut is very important to our community economy as well. And so is crab. […] But last year there was no halibut fisheries. […] And I heard there's no fishing season again this year. And if there's no fishing season, there's no halibut for us to eat.”*—Adult #3
Cultural sustainability	3	3	3	9	*“There are people here that are trying to bring it [Unangam tunuu] back, you know, and they're, you know, they're making a difference, I would say.”*—Adult #6
Food sovereignty and security	0	6	2	8	*“I like to always think of that comparison of how our people lived and what they perceived and the honor they gave and using the entire animal. And that's food security.”*—Adult #7
Advocacy for change	0	4	3	7	*“We keep going back to the meetings of, […] North Pacific Fishery Management Council and we talk about […] cutting back [bycatch]. So hopefully, you know, things will start working out.”*—Elder #2
Innovation and adaptability	1	4	2	7	*“Maybe someone should start farming, you know. […] That might be something to look into actually.”*—Adult #4
Practical engagement with subsistence	4	2	1	7	*“It's just. I’m bored. Honestly. I like hunting. If I really wanted to connect to my spiritual roots, I'd build myself a spear and a kayak and go get a seal.”*—Youth #1
Resilience	0	3	1	4	*“I would wish they would make it like the old days.”*—Elder #5
Neutrality toward food choices	1	0	0	1	*“I don’t care what people eat.”*—Youth #5
Relational value					
Knowledge transmission and intergenerational learning	4	8	5	17	*“It's like, there's a certain feeling to it when I get to do this [sealing]. This tradition still lives on and I'm the next generation to teach it to my kids […] or my grandkids.”*—Youth #2
Community cohesion, collaboration and support	5	7	5	17	*“It's not the taste. Kind of a taste. It has to do with the taste, but also just the experience I had with my family.”*—Youth #2
Cultural relations	3	8	3	14	*“Oh my gosh, it's [hunting] everything. It's us, our identity, how we grew up, how we subsisted off the land.”*—Elder #1
Food relations	2	7	4	13	*“Mh, tastes like home. […] It's a type of soul food, you know, it's the food around here, even though it might not be healthy, people love it. It speaks to the heart.”*—Adult #5
Community responsibility and provision	2	6	3	11	*“Since I have my own boat, it kind of feels like I have no choice but to provide.”*—Adult #6
Environmental relations	1	4	3	8	*“I just feel connected with nature when I do it [sealing].”*—Youth #2
Leadership and advocacy	0	2	4	6	*“A great hope I have is that we can be leaders of a resistance. Of protecting our waters, like physically. Going out in boats or blockading, getting in the ways of these trawlers. I see that as a non-violent direct action. […] And so I feel like we as Indigenous people would need to lead that.”*—Adult #9
Empowerment, emancipation and gender equality	0	2	1	3	*“I'm sure the women did the cooking and stuff, back then. But so when I started to partake in the harvest, […], I was really proud. Like, I'm a female. I could probably skin the seal faster than he could. […] And so I definitely carry that pride.”*—Adult #3

Numbers indicate the number of interviews in which the respective value was coded. The order of the events corresponds to the total count. For value definitions, see Appendix F

### Shared community history of the St. Paul Island food system

**Fig. 4:**
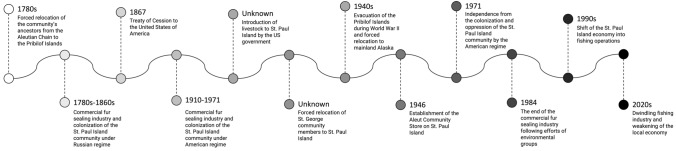
Timeline of the historical events in the St. Paul Island food system identified by this study’s interviewees

In the following, we summarize the history of St. Paul Island based on the historical events identified in the narrative interviews (Fig. 4). Numbers indicate the number of interviews in which the respective event was coded.

The Pribilof Islands’ history is unique among other Arctic communities in that their inhabitants originally belonged to the Indigenous peoples of the Aleutian Islands, but were forcibly taken from their homes by Russian colonizers and brought to the—at that point uninhabited—Pribilof Islands (St. Paul and St. George) in the late 1780s to harvest fur seals to meet the growing demand for fur in global markets (*n* = 2) (Yesner 2017; Black 1999). After decades of commercial fur seal harvesting under Russian control (*n* = 8), the USA bought Alaska in 1867 and eventually took over the fur seal industry (*n* = 13). With the change of colonial power, the Aleut community on the Pribilof Islands experienced a further deterioration of their working and living conditions. Intervening in their culture, marriages, movement to and from the islands, employment, and administration of justice, the Aleuts were now classified as wards of the US government, which included that they were paid in kind for their work in the seal harvest, and experienced repression and discrimination by government agents. The US government also rationed the Aleuts' food and later even introduced livestock to St. Paul (*n* = 3) (Jones 1980). In an attempt to merge the populations of St. George and St. Paul Island for the federal seal harvesting and fox trapping efforts, many residents of St. George were transferred to St. Paul Island (*n* = 1). After a Japanese attack on the Aleutian Islands during World War II in 1942, US government officials once again forcibly relocated Alaska Natives from the Aleutian and Pribilof Islands (*n* = 5). The local community was forced to inhabit internment camps, which were merely abandoned canneries throughout Southeast Alaska, where lack of food, housing, heat, and medicinal care resulted in deaths and trauma for many (Kohlhoff 1995). After the war, many community members returned to the Pribilof Islands, where, in 1946, the Aleut Community Store was established (*n* = 4). The store was the primary source of income for the local tribe for many years and financed the fight for Aleut independence (*n* = 4). It was only in the mid-1960s that the Pribilof Aleuts gained full rights as American citizens (Torrey and Krukoff 1978). With the passage of the Alaska Native Claims Settlement Act (ANCSA) in 1971, assimilation policies continued with a corporation structure implemented and monetary settlements issued to compensate Indigenous peoples across Alaska for the taking of land (Torrey and Krukoff 1978). Following these land claims, the commercial fur seal harvest was finally managed by the Aleuts themselves. However, in response to decades of excessive harvesting and the demands of a growing environmental and animal rights movement in the 1970s and 1980s, the last commercial fur seal harvest occurred in 1984 on St. Paul Island. What was favorable for the, by the time, substantially depleted local fur seal populations once again deprived the community of its livelihood (*n* = 7). During these difficult years, the Aleut Community Store was also transferred to the Alaska Commercial Company. After considerable lobbying, the islanders eventually received compensation from the United States to diversify their economy and develop their fisheries (*n* = 2). What followed were years of economic upswing in which the crab and halibut fishing industry primarily supported the economy of St. Paul. In October 2017, the store was retransferred to the TGSPI and is currently managed and operated by the tribe. The industrial global pollock fishery (Lyons et al. 2016), their high bycatch rates, and the climate crisis have more recently affected local halibut and crab fishing, drastically weakening the local economy (*n* = 2) (Fig. 4). A tabular overview of all historical events mentioned is given in Appendix D.

### Perceived impact of past events and current factors on the present and future food system

**Fig. 5:**
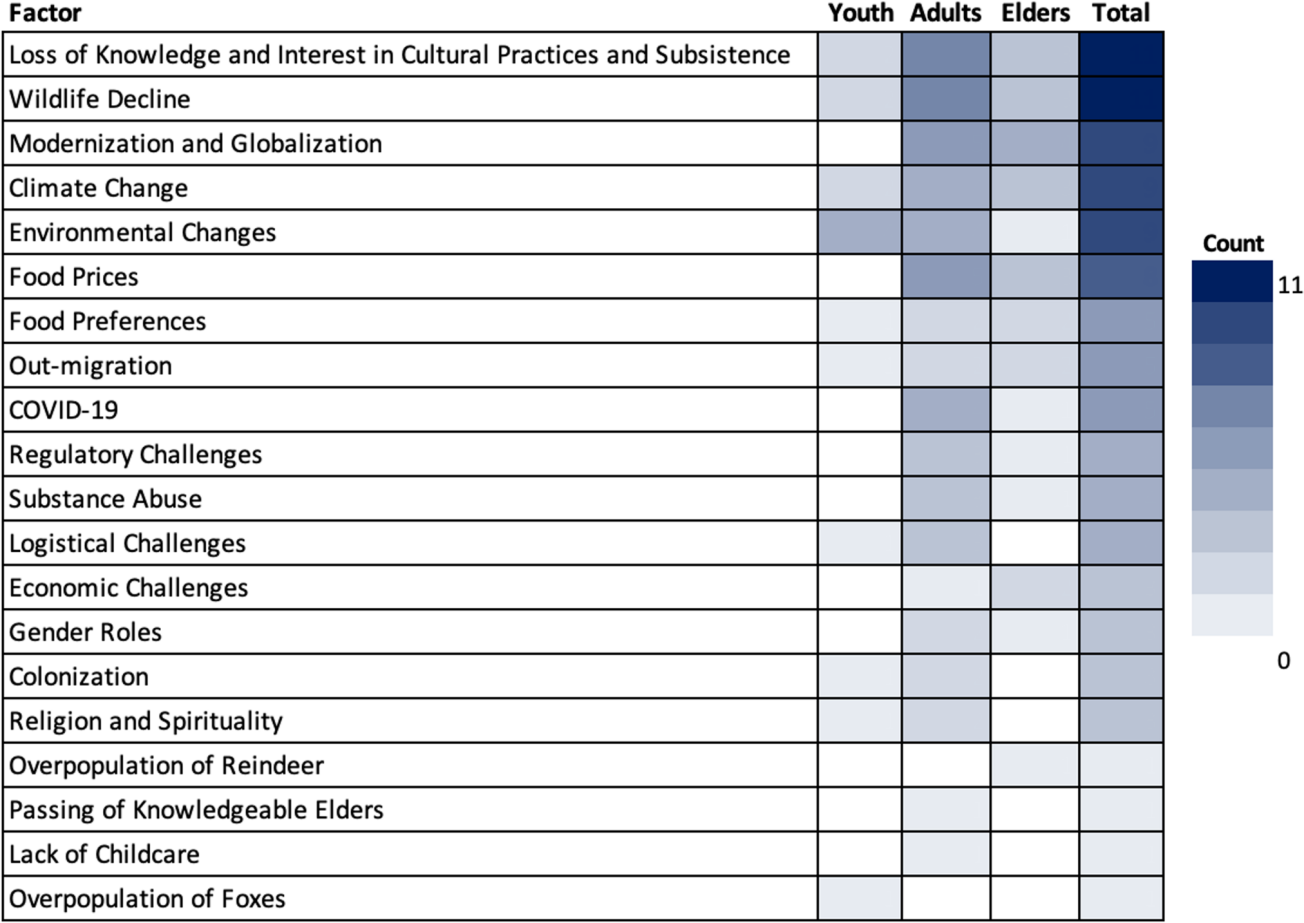
Factors that determine and shape the food system today and in the future, according to the study’s interviewees. Colors indicate the number of interviews in which the respective factor was coded

According to the interviewees of this study, both the continuing effects of past events and new factors determine the present and future food system on St. Paul Island. The long-lasting effects of the colonization and oppression of the St. Paul Island community by the Russian and American regimes (*n* = 3), as well as modernization and globalization, including lifestyle changes and the introduction of modern technologies (*n* = 9), were mentioned to affect the food system in various ways. The loss of knowledge and interest in cultural practices and subsistence activities (*n* = 11) and changing food preferences and dietary choices (*n* = 5) were mentioned most often. Furthermore, study participants discussed influencing factors regarding both the subsistence and cash-based sides of the local mixed economy. From the interviewees’ perspective, climate change (*n* = 9), environmental changes (*n* = 9), and wildlife decline (*n* = 11) notably impact subsistence harvesting and the sharing of local resources. The passing of knowledgeable Elders in the community (*n* = 1) and the changing values placed on religion and spirituality (*n* = 3) are other factors mentioned in this regard. With respect to the cash-based side of the food system, high food prices (*n* = 8), as well as various logistical (*n* = 4), regulatory (*n* = 4), and economic challenges (*n* = 3), were brought forward as impacting the food system. The interviewees often linked these challenges to out-migration (*n* = 5) and substance abuse (*n* = 4). Other factors mentioned are COVID-19 (*n* = 5), changing gender roles (*n* = 3), the lack of childcare (*n* = 1), and the overpopulation of reindeer (*n* = 1) and foxes (*n* = 1) (Fig. 5; Appendix E).

### Local food system values

We identified six intrinsic values across all interviews (Table 1). Apparent in the majority of the interviews is the acknowledgment of historical influences on the St. Paul Island food system (*n* = 16) and a deep appreciation and respect for the traditional Aleut culture (*n* = 15). Most interviewees also mentioned their deep respect for their natural environment (*n* = 11) and awareness of the changes within (*n* = 15). Additionally, some interviewees placed value on a strong work ethic (*n* = 10) and voiced their preference for authentic food sources due to their intrinsic value (*n* = 8).

We found 14 instrumental values across all interviews (Table 1). Thereof, the good taste of certain foods (*n* = 16) and the value placed on community support and sharing of foods for one’s food security (*n* = 15) were mentioned by most interviewees. The majority of interviewees also placed value on the fun and joy derived from engaging in subsistence activities (*n* = 13) and on the autonomy and independence they gain through self-sufficiency (*n* = 11). Other reoccurring instrumental values were the sustainability of the environment (*n* = 10), the economy (*n* = 10), and culture (*n* = 9) for the sustenance, survival, and security of the St. Paul Island community in current and future generations, as well as the health benefits derived from certain dietary choices (*n* = 10).

Lastly, we identified eight relational values across all interviews (Table 1). Most interviewees placed value on the transmission of ILTK and intergenerational learning opportunities (*n* = 17) and on community cohesion through relations and sharing (*n* = 17). Many also emphasized the importance of their relations to Aleut culture (*n* = 14), food (*n* = 13), and the environment (*n* = 8) through engagement with traditional practices, the consumption of certain foods, or with nature, respectively. The majority of interviewees shared a sense of responsibility toward the provision and sharing of subsistence foods in the community (*n* = 11). Other relational values mentioned were the local Indigenous leadership and advocacy of community rights (*n* = 6) and the awareness and appreciation of changing gender stereotypes, including the increasing role of women in the food system (*n* = 3).

## Discussion

We aimed to contribute to understanding the role of values as deep LP for transformative change in mixed Indigenous food systems through a case study on St. Paul Island. Through storytelling sessions with ACSPI youth, non-Elder adults, and Elders, we identified events and factors that our interviewees perceived to have shaped or shape the food system and diverse food system values. We found historical events expressed in the stories of most interviewees, indicating the continued presence of the past in their present consciousness (objective (i)). We found the majority of factors identified to affect the present and future food system to be food system challenges that threaten the culture, food sovereignty, and food security of the community and hinder ACSPI members from acting upon the values they hold in the food system (objective (ii)). Lastly, we found values in the St. Paul Island food system to be a diverse mix of intrinsic, instrumental, and relational values, with often plural values attributed to specific food system elements (objective (iii)). In the following, we discuss these results in the context of intergenerational trauma and the value change debt to enrich our understanding of the role of values as deep LP for transformative change.

### Plurality of values reflecting a mixed food system

The ACSPI carries a complex history characterized by various traumatic events and experiences, so local stories can be a way of processing the past and present and creating a shared understanding by communicating them across generations (Iseke 2013). Values delineated from the story transcripts are a diverse mix of intrinsic, instrumental, and relational values (Table 1) relating to valuations of both traditional and new elements in the local food system (objective (iii)). Hence, the diverse value system reflects the nature of the mixed Indigenous food system on St. Paul Island, where a cash-based wage economy is combined with subsistence harvesting and sharing resources (Zimmermann et al. 2024; Ready and Power 2018). Within this value system, a pluralistic valuation of the food system is seen. Hence, the coexistence of multiple intrinsic, instrumental, and relational values allows for a multiplicity of perspectives that support collective and reflexive value formation processes (Himes and Muraca 2018).

Such a mixture of values that relate to both traditional and new food system elements is also seen in other mixed food systems in Arctic (Bogdanova et al. 2020; Hillmer-Pegram 2016; Ready and Power 2018) and non-Arctic (Utami et al. 2022; Milcu et al. 2014) contexts. This value pluralism can lead to conflicts between values, which can hinder integrating them equally into decisions (Kenter et al. 2019). Historically, economic values have been favored in policy and decision-making (Demaria 2010), while traditional Indigenous values have been ignored (Martinez-Alier 2013). Our study contributes to the increasing evidence that such power conflicts require researchers, practitioners, and decision-makers to embrace the plurality of values to equally integrate mixed values into decisions and maximize benefits for multiple stakeholders (Arias-Arévalo et al. 2017; Belisle-Toler et al. 2021; Kenter et al. 2019).

### Cultural resilience in the face of trauma

The ACSPI shares a challenging history in which several major events have disrupted their food system (Fig. 4) (Sepez et al. 2007; Black 1999; Kohlhoff 1995; Jones 1980). These historical events are embedded in local food system stories across the three generations in the ACSPI that participated in this study, confirming the continued presence of the past in the collective local consciousness (Appendix C). The unresolved nature of past events means the trauma experienced by past generations (Elders) still affects their descendants (non-Elder adults, youth) (Crawford 2014). Subsequently, these traumatic events are still very influential in the present food system (Fig. 5; Appendix E), as the present is continuously being informed and shaped by past experiences (Antrop 2005). At the same time, new challenges emerge in the present that are expected to influence the future food system further (Fig. 5; Appendix E).

Despite acute disturbances that produce ongoing and unresolved trauma and significant changes in the local food system (Zimmermann et al. 2024), values that relate to traditional Indigenous livelihoods, such as a deep respect for the local culture and environment, community support and sharing, and social–ecological relations, remain central to the ACSPI value system (Table 1). These traditional Indigenous values^1^ are important sources of cultural continuity, which, in Indigenous communities, is deeply intertwined with community resilience (Berliner et al. 2011). Indigenous concepts of resilience are grounded in values that have persisted despite changes in the nature of community life (Kirmayer et al. 2011a). Indigenous culture across the Arctic has generally been resilient in the face of colonialism and other hardships (Poppel 2017). Research suggests that narratives of historical continuity provide resources for resilience in Indigenous communities as they confront the ruptures that have occurred in the past and support identity building, emotion regulation, problem-solving, social positioning, and communal solidarity (Kirmayer et al. 2011b). Narratives of cultural continuity, hence, encourage the perpetuation of cultural traditions and identities and offer pathways for cultural revitalization, making them essential for the overall resilience of Indigenous communities. Associated with a sense of heritage and shared purpose across time and generations (Salusky et al. 2021), cultural continuity has also been positively linked to Indigenous health and well-being, such as shown in the context of suicide rates among Indigenous youth in Canada (Newell et al. 2020; Ulturgasheva et al. 2014).

However, understandings of culture are shaped by historical experiences and modified through time (Wexler 2014). Different generations have experienced varying levels of oppression and marginalization, and how individuals make meaning and take strength from particular notions of culture consequently differs across generations (Wexler 2014; Wexler et al. 2014). Owing to these changes through time, generational inclusivity is essential for understanding perceptions of the past, present, and future and how these relate to desired futures (Marschütz et al. 2020; Herman-Mercer et al. 2016). Elders embody cultural continuity and are central to younger generations’ acquisition of traditional knowledge linked to their ancestors (Salusky et al. 2021). Non-Elder adults and youth play an important part in safeguarding cultural continuity as they link the past as transferred to them via storytelling and the present through experiential learning and, ultimately, shape the future as they age. The events and values obtained from the stories shared by our interviewees hence express individual perspectives and opinions, but when added to the collective, traditional Unangax̂ values remain central for all (APIA 2024). Nonetheless, as the food system changes and new challenges emerge (Fig. 5; Appendix E), values reflecting modern food system elements, such as economic sustainability, innovation and adaptability, and emancipation and gender equality, stand alongside traditional Indigenous values in the contemporary ACSPI value system (Table 1). This development often fosters diverse and plural valuations in the food system but also bears the potential of gradually eroding traditional Indigenous values and, hence, poses a risk to cultural continuity (Riechers et al. 2020; Wahab et al. 2012).

### The value change debt as a window of opportunity for transformative change

Values relate to deep system levels (Abson et al. 2017; Meadows 1999) and, as such, are strong LP for transformative change, as how humans perceive and value the world fundamentally shapes their actions and behavior (Lam et al. 2020; West et al. 2020). Although the St. Paul Island food system has changed significantly over the time of permanent inhabitance (Appendix G), important traditional Indigenous values persist in the local community that relies on the local food system. This time lag between changes in peoples’ held values following changes to the system around them has previously been described as a value change debt and is equivalent to the concept of extinction debt in ecology (Horcea-Milcu et al. 2017). In rural Romania, it was found that rural communities held onto traditional rural values despite the landscape having undergone significant change and intensification (Horcea-Milcu et al. 2017). Yet, fundamental system changes have been shown to erode traditional values over time in other contexts (Riechers et al. 2020; Wahab et al. 2012). Similarly, in the ACSPI food system, the legacies of historical events, in combination with new systemic changes, are partly preventing the community from expressing their traditional Indigenous values, a concern clearly articulated in the following quote by research participant Youth #2:

It definitely changed our food system because introducing American foods, lactose, milk and stuff, which we did not have. And our bodies are still not used to it, even to this generation. It changed our way of life. We used to be, we used to live off the land and we used to like [the food] the land provided us, we provided the land. And now our land has just [changed], we don't do that anymore. The food here is not even good. Prices are too high. It's hard to get stuff here. Fresh produce is horrible. Because of the Americans, because they colonized us. And it's just such a chain reaction where it just it's all falling.—Youth #2

We argue that this value change debt represents an important window of opportunity in the case of the St. Paul Island food system, and potentially in other contexts, in which to reduce the system elements that currently prevent the community from acting upon its held values to create a food system inherently designed to embrace these values. Leveraging the value change debt means intentionally designing and implementing interventions to the food system that allow the community to live out its values more fully without having to change community values, an undertaking that is considered extremely difficult (Meadows 1999). For instance, if the community places high value on sustainability, self-sufficiency, and cultural preservation yet faces barriers such as regulatory challenges or economic constraints that limit their ability to engage in traditional practices, targeted interventions should aim to reduce these barriers. This tension is described in the following quote by research participant Non-Elder adult #9:

We've had attempts at having a local reindeer being made available for purchase, [but] there's […] things with the FDA [Food and Drug Administration] needing to approve them.—Non-Elder adult #9

By acknowledging and addressing the system elements that currently inhibit the expression of community values, there is potential to reconfigure the food system in a way that respects Tribal sovereignty and integrates these values and enhances the system's sustainability, resilience, and relevance to the community's way of life, an idea clearly reflected in the following quote by research participant Elder #5:

I wish they would make it like the old days. When I was growing up, they had two movie theaters here and they had like five coffee shops. And it was a lot of things to do. We didn't have internet, we didn't have TV, you didn't even have phones. So we were out outdoors all the time. And now you don't see anybody out hardly anymore. They're playing a TV game, or watching TV, playing games. Yeah. That's not good.—Elder #5

In essence, the value change debt underscores the untapped potential within the ACSPI to reshape its food system in alignment with its enduring cultural values and aspirations. Leveraging this window of opportunity requires a concerted effort to understand the specific barriers to value expression within the food system and to implement changes that enable the community to bridge the gap between their values and their lived reality, thereby creating a food system that is inherently more aligned with and supportive of their cultural identity, needs, and aspirations.

### Outlook

With the empirical example presented in this study, we contribute to the often fuzzy field of values literature (Kenter et al. 2019) by showing how values can inform and lead to decision-making and action-taking. Integrating values-driven community data into the design and implementation of food system interventions can enhance their acceptance and effectiveness, aligning solutions more closely with community priorities and cultural practices, facilitating adaptation, and strengthening resilience over time (Berliner et al. 2011). However, future research could benefit from combining this deeper system level, values-driven community data with data on other system levels (parameters, feedbacks, design) (Charnley et al. 2017; Abson et al. 2017) to plan effective interventions, an approach that has already proven promising in Indigenous health research (Donatuto et al. 2014; Gregory et al. 2016) and climate change adaptation planning (Reid et al. 2014). Another promising avenue for future research is a more nuanced analysis of generational differences in values. While our focus was on gaining a representative sample of values in the community across generations, it could be interesting to analyze in more depth what differences exist among generations. For example, we found that although youth, non-Elder adults, and Elders mentioned similar events, factors, and values, their descriptions differed greatly in scope and detail. Whereas youth tended to use fewer words and mention things more implicitly, non-Elder adults and Elders shared more detailed perspectives. Future studies might also benefit from examining value differences per gender category (Hoogensen Gjørv 2017) and season (Huet et al. 2017).

#### Implications of findings for the larger research project

In this study, we examined the potential role of values as deep LP for transformative change in the context of their historical co-evolution with the mixed Indigenous food system on St. Paul Island. This focus is borne out of a larger research project focused on understanding how to leverage a transformation in the St. Paul Island food system to increase local food security (Appendix A). The findings presented in this study, namely that there is a mismatch between the diverse values community members hold and the reality of the food system (Horcea-Milcu et al. 2017), have led to the realization that the current food system does not allow community members to act and live fully according to their values. This understanding has informed participatory scenario planning and pathways development workshops with the ACSPI and their TGSPI to co-design a shared community vision of the future food system based on intergenerational community values. As a part of this process, five actionable pathways were co-developed along with specific interventions that can be implemented locally to address current barriers to value expression and initiate a transformation in the St. Paul Island food system. Specifically, the co-designed interventions, such as the formation of a Community Council and the creation of a farmer’s market, aim to increase local capacity building, economic diversification, local agency, subsistence lifestyles, community cohesion, and health to align the food system better with the intergenerational values held therein. Alongside these interventions and recognizing the power of stories to transport and strengthen community values, the Tipping the Iceberg podcast has been designed as a narrative-based intervention to foster the dissemination of positive narratives about the St. Paul Island food system (Zimmermann and Aleut Community of St. Paul Island 2024–present).

## Conclusions

Despite major disturbances that produce ongoing and unresolved trauma and significant changes in the local food system, traditional Indigenous values remain central in the ACSPI values system. The legacies of past events and new emerging factors hinder local community members from manifesting their values through engaging in cultural practices. The research presented in this study indicates a value change debt, i.e., a time lag between changes in peoples’ held values following changes to the system around them, as a window of opportunity for transformative change in which to break down the system elements that currently prevent local communities from expressing their held values to create a food system inherently designed to embrace these values.

Our findings provide valuable insights for future research endeavors that aim to design and implement effective interventions in (Indigenous) food systems to leverage transformative change and enhance food security. The direct benefit of this inquiry to the local community in this study is that we defined the values community members currently hold in the food system and identified the mismatch between these values and the reality of the local food system. This observation led to the realization that the current food system does not allow community members to act and live fully according to their values. Our analysis lays the foundations for the design of food system interventions that steer the food system in a direction that makes it possible to live according to these values (and thus, for example, allows increased participation in subsistence practices). The ACSPI will likely benefit from interventions to the food system that uphold Tribal sovereignty and support reconnection with traditional cultural practices to reinforce local values and ultimately support individual and collective community healing.

## Supplementary Information

Below is the link to the electronic supplementary material.

Supplementary file1 (DOCX 54 KB)

## Footnotes

1 We use the term ‘traditional Indigenous values’ in this paper to refer to values relating to traditional Indigenous livelihoods and food system elements. In the context of the study of the St. Paul Island food system, our research partners and participants often used the term ‘traditional’ to refer to Unangax̂ values and ways of living. Yet, we acknowledge the increasing body of literature that critiques the dichotomy of ‘traditional’ and ‘modern’ in (post-)colonial contexts (i.e., see Smith 2021; Mingolo et al. 2002; Porsanger 2011; Germond-Duret 2016).
