# Supplementary material for: Value change debt as a window of opportunity for transformative change: a case study on the mixed Indigenous food system of St. Paul Island, Alaska
Source: Sustain Sci. 2025 Mar 21;20(5):1883–901. doi: 10.1007/s11625-025-01665-z (PMC12414080; doi:10.1007/s11625-025-01665-z)
Supplement: Supplementary file 1 — Supplementary file1 (DOCX 54 KB) [file 11625_2025_1665_MOESM1_ESM.docx]

**Supplementary material**

**Appendix A.** Description of the Tipping the Iceberg research project.

The research presented in this paper has been designed as part of the four-year Tipping the Iceberg research project that combines complex systems science with transdisciplinary approaches to leverage a sustainable food system transformation on St. Paul Island. The Tipping the Iceberg research project is a collaboration between Utrecht University, the Aleut Community of St. Paul Island Tribal Government (TGSPI), and the Aleut Community of St. Paul Island (ACPSI) that aims to identify effective leverage points in the mixed local food system and initiate pathways toward a future of increased food security for the community. The project consists of several work packages, which are designed and implemented in ongoing consultation with TGSPI and ACSPI representatives. The overarching framework in this project is the leverage point (LP) framework, which suggests that in complex systems, there are certain points at which interventions are particularly effective and can lead to transformative change (Meadows 1999). The LP framework recognizes systems across their four levels of systemic depth (parameter, feedback, design, and intent) and generally considers interventions at deep system levels to be more effective in fostering transformative change than interventions at shallower levels (Abson et al. 2017). While previous work packages of the Tipping the Iceberg project focused on shallower system levels (Zimmermann et al. 2024), the work package whose findings are presented in this paper aims to understand the deep intent level of the St. Paul Island food system. With all findings combined, the project seeks to gain a holistic understanding of the social-ecological food system to identify interdependencies and feedback between system levels and ultimately find important leverage points for a sustainable transition. Based on this understanding of the St. Paul Island food system, the researchers facilitate several workshops with ACSPI and TGSPI representatives later in the project to co-create a shared community vision of the future food system and identify effective pathways toward a more sustainable system state. Specifically, the realization resulting from the findings presented in this paper, namely that the current food system does not allow community members to act and live fully according to the values they hold in the food system, informed the co-design of five actionable pathways along which the ACSPI and their TGSPI can implement specific interventions to initiate a transformation in the St. Paul Island food system locally.

Across all work packages, the researchers take a highly participatory approach that involves ACSPI members and TGSPI representatives in various ways and empowers them to bring in their hopes and ideas for a food-secure food system on St. Paul Island. Thus, the Tipping the Iceberg research project provides a unique opportunity to assess whether complex systems science combined with transdisciplinary approaches can lead to actual changes on the ground.

**Appendix B.** Positionality statement of the author team.

The team of researchers for this study comprises interdisciplinary academic backgrounds and stages, as well as different levels of engagement and familiarity with the St. Paul Island community. The Aleut Community of St. Paul Island (ACSPI) is a title by which the federal government of the United States formally recognizes ‘the tribe’ residing on St. Paul Island. The Aleut Community of St. Paul Island Tribal Government (TGSPI) is a governmental venue through which the Unangan of St. Paul Island can fulfill their intrinsic rights and responsibilities, and support, recollect, practice, and pass on their culture. The TGSPI promotes, maintains, and protects cultural practices, awareness, preservation, self-governance, and self-determination for the ACSPI. Many ACSPI members and TGSPI representatives have contributed in various ways to the Tipping the Iceberg research project. For the research presented in this paper, contributions by ACSPI members and TGSPI representatives include giving interviews and sharing stories about the food system, constant dialogue and exchange, advisory activities, and sharing knowledge, skills, and resources with the co-authors.

Silja Zimmermann is a white, non-Indigenous female from Germany who is the PhD candidate of this project with a background in geography and conservation ecology and a specific focus on Arctic SES. For the research presented in this paper, she spent three months in the community in March, April, and May 2022 and carried out the data collection with the help of a local research assistant (KLZ). She spent another three months in the community in August and September 2023 and April 2024 to continue working on other parts of the Tipping the Iceberg research project. Silja analyzed the data and wrote the first version of the manuscript, which she then revised based on input from all co-authors.

Brian Dermody is a white, non-Indigenous male of Irish nationality who has lived in the Netherlands for the last 14 years. His interest in this research arises from a passion for finding ways to co-develop solutions for sustainability based on integrating Western scientific complex systems understanding with non-academic knowledge of how stakeholders with high-interest experience such systems. He feels a combination of objective and subjective understanding is crucial to creating action for just and sustainable systems. For the research presented in this paper, he helped conceive the idea and provided input on research design and data analysis. He reviewed earlier versions of the manuscript and read and approved its final version.

Courtney Carothers is a white settler scholar of northwestern European ancestry based in Dgheyey Kaq’ or Anchorage, Alaska. She has devoted her career to working with fishing communities across Alaska to better understand the social and cultural dimensions of fishery systems and to improve education, research, and governance processes to better include these dimensions. She partners with Indigenous communities to promote social and environmental justice goals. For the research presented in this paper, she helped conceive the idea, reviewed earlier versions of the manuscript, and read and approved its final version.

Valeria Di Fant is a white, non-Indigenous female from Italy who has lived in the Netherlands for the last seven years. Her background is in sustainability and climate science, and she is a PhD student currently researching adaptation pathways and the potential for these to plan for transformative climate adaptation. She contributed to the methodological development of this research while working as a junior teacher at Utrecht University’s Copernicus Institute of Sustainable Development. Valeria also reviewed earlier versions of the manuscript and read and approved its final version.

Lauren Divine identifies as a white, middle-class American female from Tybee Island, Georgia, the ancestral homelands of the Mvskoke (Muscogee), Yamasee, and Guale. She has had the privilege to live and work as a guest on Unangax̂, Dënéndeh, Tanana, and Dena’ina lands and waters for over ten years. She recognizes that her identity and positionality can influence all aspects of [research](https://en.wikipedia.org/wiki/Research), including the questions, [study](https://en.wikipedia.org/wiki/Scientific_method) design, [data](https://en.wikipedia.org/wiki/Data) collection, and [data analysis](https://en.wikipedia.org/wiki/Data_analysis). She strives to operate respectfully through an Indigenous lens to realize positive benefits to the Indigenous communities she works with.

Veronica Padula is a white, non-Indigenous female researcher who has been employed by the Aleut Community of St. Paul Island (ACSPI) since 2017. In their roles with ACSPI, VP and LD have collectively spent 18 years fostering relationships with tribal and community members through various research and education activities, including community-engaged research centering TILK of the St. Paul Island ecosystem and building capacity for local Indigenous youth to lead and participate in research. For the research presented in this paper, LD and VP facilitated the ongoing exchange between the ACSPI, their Tribal Government, and the other co-authors to enable the co-design of the research process. Both also reviewed earlier versions of the manuscript and read and approved its final version.

Kadyn Lestenkof-Zacharof is an Unangax̂ woman who has grown up on Tanax̂ Amix̂ (St. Paul Island) her whole life. She assisted with the data collection for this research. Her goals are set on marine and environmental protection, sustainability, and conservation.

Bert Theunissen is a white, non-Indigenous male researcher from the Netherlands with a background in biology and the history and philosophy of science. He is particularly interested in the social and cultural construction and circulation of knowledge. For the research presented in this paper, he helped conceive the idea, reviewed earlier versions of the manuscript, and read and approved its final version.

Martin Wassen is a white, non-Indigenous male researcher from the Netherlands with a background in environmental sciences. For the research presented in this paper, he helped conceive the idea, reviewed earlier versions of the manuscript, and read and approved its final version. Additionally, he spent two weeks on St. Paul Island in September 2023 for a later part of the wider research project not presented in this paper.

Ine Dorresteijn is a white, non-Indigenous female researcher from the Netherlands with a sustainability science and social-ecological systems background. In this research project, she recognizes the complexities inherent to the diverse perspectives and various forms of knowledge of Arctic Indigenous food systems. She strives to respectfully contribute to the co-creation of knowledge for just and resilient food systems and is aware of the inherent bias embedded within her societal positionality and research lenses. For the research presented in this paper, she helped conceive the idea and provided input on research design and data analysis. She reviewed earlier versions of the manuscript and read and approved its final version. Additionally, ID spent two weeks on St. Paul Island in September 2023 and three weeks in April 2024 to support SZ in preparing and facilitating other research for the Tipping the Iceberg project. She also spent two summers on St. Paul Island to study the impacts of climate change on seabirds in 2008 and 2009.

While our collective experiences as academics carry inherent biases and perspectives, we are deeply conscious of our outsider status in the Alaskan Indigenous context. We have continuously strived for cultural sensitivity, reflexivity, and collaboration, recognizing the need to center the voices and expertise of the St. Paul Island Aleut community in our work.

**Appendix C.** Interview guide for the narrative interviews conducted in storytelling sessions.

**Interview details**

Date: __________

Time: __________

Name: __________

Gender: __________

Age: __________

Location: __________

Note: __________

**Topic guide**

- Stories and narratives about food/subsistence activities/the food system
- The importance of stories and narratives around food for the individual and the St. Paul Island community as a whole
- Past events and circumstances that have determined and shaped the food system
- Present events and circumstances that determine and shape the food system
- Future events and circumstances that will determine and shape the food system
- Values and attitudes towards food/subsistence activities/the food system

1. **Initiation phase: General questions**

- Who are you?
- Where do you come from?
- Who is your family?
- What is your role in the community?
- What is your role in the local food system?
- Which activities are you involved in that are related to food?

1. **Main narration: Main questions**

- Can you tell me a story about something that has to do with food? (*individual*)
- What are some of the most important stories about food that you remember being told on the island? (*collective*)

**(c) Questioning phase 1: Follow-up questions**

- Is this all you want to tell me?
- Is there anything else you want to say?
- What happened before/after/then?

**(c) Questioning phase 2: Topic guide questions**

- Are there any stories you can think of that you have been told by your parents or grandparents related to food?
- Are there any stories you can think of that you are telling yourself related to food?
- What do you think is the role of stories in your community in the context of food?
- What do you think were the circumstances that determined the food system in the past?
- Can you think of any past events that have shaped the food system as it is today?
- What do you think are the circumstances that determine the food system today?
- Why do you think that has changed?
- What do you think are the circumstances that will determine the food system in the future?

1. **Concluding talk**

- Why did you tell that story?

**Rules for the researcher**

- The researcher (listener) must behave respectfully towards the interviewee (storyteller).
- The listener does not interrupt the storyteller and only asks follow-up questions after they have finished their story.

**Appendix D.** Historical events that have determined and shaped the food system in the past, according to the study’s interviewees. Numbers indicate the number of interviews in which the respective event was coded. The order of the events corresponds to the chronological timeline.

| **Historical Event** | **Definition** | **Youth (n=5)** | **Adults (n=9)** | **Elders (n=5)** | **Total Count** |
| --- | --- | --- | --- | --- | --- |
| Relocation of forefathers to Pribilof Islands | The relocation of the community’s ancestors from the Aleutian Chain to the Pribilof Islands | 0 | 1 | 1 | 2 |
| Colonization by Russians | The colonization and oppression of the St. Paul Island community by the Russian regime | 1 | 7 | 0 | 8 |
| Colonization by US Government | The colonization and oppression of the St. Paul Island community by the American regime | 2 | 7 | 4 | 13 |
| Introduction of Livestock | The introduction of livestock to St. Paul Island by their colonizing regimes | 0 | 1 | 2 | 3 |
| Independence from US Government | Gaining independence from the colonization and oppression of the St. Paul Island community by the American regime | 0 | 2 | 2 | 4 |
| Evacuation WWII | The evacuation of the Pribilof Islands during World War II and the correspondent relocation of the Pribilof Island communities to canneries in southeast Alaska | 0 | 2 | 3 | 5 |
| Relocation from St. George Island to St. Paul Island | The forced relocation of community members from St. George Island to St. Paul Island | 0 | 1 | 0 | 1 |
| Commercial Fur Seal Industry | The commercial fur sealing industry under Russian and American regimes | 2 | 1 | 4 | 7 |
| Shift from Fur Seal Industry to Fishing Economy | The end of the commercial fur seal industry and the following shift of the local economy into fishing operations | 0 | 0 | 2 | 2 |
| Dwindling Fishing Industry | The decline in halibut and crab fishing and the resulting weakening of the local economy | 0 | 2 | 0 | 2 |
| Appearance of the Aleut Community Store | The appearance of the Aleut Community Store on St. Paul Island | 2 | 1 | 1 | 4 |
| Gender Roles | Traditionally defined gender roles with men primarily being the hunters and providers and the majority of women cooking and staying at home | 0 | 2 | 1 | 3 |

**Appendix E.** Factors that determine and shape the food system today and in the future, according to the study’s interviewees. Numbers indicate the number of interviews in which the respective factor was coded. The order of the events corresponds to the total count.

| **Factor** | **Definition** | **Youth (n=5)** | **Adults (n=9)** | **Elders (n=5)** | **Total Count** |
| --- | --- | --- | --- | --- | --- |
| Loss of Knowledge and Interest in Cultural Practices and Subsistence | The loss of Indigenous, Traditional, and Local Knowledge (ILTK) and interest in cultural practices and subsistence activities | 2 | 6 | 3 | 11 |
| Wildlife Decline | The decline of subsistence species and other wildlife populations due to various reasons, including overhunting, overfishing, or climate change | 2 | 6 | 3 | 11 |
| Modernization and Globalization | Modernization and globalization tendencies, including lifestyle changes, generational changes, and the introduction of modern technologies (i.e., internet, gaming, social media) | 0 | 5 | 4 | 9 |
| Climate Change | The effects of climate change, including warming temperatures and changing weather patterns | 2 | 4 | 3 | 9 |
| Environmental Changes | The effects of broader environmental changes, including pollution, changes in subsistence species, and coastal erosion | 4 | 4 | 1 | 9 |
| Food Prices | The high costs for food at the Aleut Community Store and the lower costs for online orders of food | 0 | 5 | 3 | 8 |
| Food Preferences | Changing food preferences of St. Paul Island community members and resulting dietary changes | 1 | 2 | 2 | 5 |
| Out-migration | The out-migration of St. Paul Island community members to mainland Alaska or the lower 48 states of America | 1 | 2 | 2 | 5 |
| COVID-19 | The effects of the COVID-19 pandemic on the St. Paul Island food system, including the closing of the fishing season, quarantine restrictions and supply chain issues | 0 | 4 | 1 | 5 |
| Logistical Challenges | Logistical barriers in the St. Paul Island food system, including weather, shipping and freezer issues | 1 | 3 | 0 | 4 |
| Regulatory Challenges | Regulatory barriers to the utilization of local food sources, including regulations by the federal government or the Food and Drug Administration (FDA) | 0 | 3 | 1 | 4 |
| Substance Abuse | The abuse of substances such as alcohol and drugs by St. Paul Island community members and the resulting issues, including the lack of engagement in subsistence activities and financial issues | 0 | 3 | 1 | 4 |
| Colonization | The long-lasting effects of the colonization and oppression of the St. Paul Island community by the Russian and American regimes | 1 | 2 | 0 | 3 |
| Religion and Spirituality | The changing value placed on religion and the spiritual connection to food and subsistence species by St. Paul Island community members | 1 | 2 | 0 | 3 |
| Economic Challenges | The high costs of living and utilities on St. Paul Island | 0 | 1 | 2 | 3 |
| Gender Roles | The change of traditionally defined gender roles and the emancipation of women in the St. Paul Island community | 0 | 2 | 1 | 3 |
| Passing of Knowledgeable Elders | The passing of knowledgeable Elders in the St. Paul Island community | 0 | 1 | 0 | 1 |
| Overpopulation of Reindeer | The potential overpopulation and expected starvation of the reindeer population on St. Paul Island | 0 | 0 | 1 | 1 |
| Overpopulation of Foxes | The potential overpopulation and expected interference with human waste of the fox population on St. Paul Island | 1 | 0 | 0 | 1 |
| Lack of Childcare | The lack of childcare on St. Paul Island and the resulting collision of care-giving work, wage labor, and subsistence activities | 0 | 1 | 0 | 1 |

**Appendix F.** Intrinsic, instrumental, and relational food system values, as identified from the stories and narrative interviews with youth, non-Elder adults, and Elders. Numbers indicate the number of interviews in which the respective value was coded. The order of the values corresponds to the total count.

| **Intrinsic Value** | **Definition** | **Youth (n=5)** | **Adults (n=9)** | **Elders (n=5)** | **Total Count** | **Quotes** |
| --- | --- | --- | --- | --- | --- | --- |
| Historical Awareness | Awareness and acknowledgment of historical influences on the St. Paul Island food system, including colonization, and the forced evacuation during World War II | 4 | 7 | 5 | 16 | *“You know, we were brought here by the Russians and we were technically enslaved, we were forced to kill seals and it was only for their fur. My assumption is that over time, my people just learned to eat it because […] that’s all they were given, the only options.”* – Adult #3 |
| Appreciation and Respect for Culture | Appreciation, respect, and preservation of Aleut culture, practices, language, traditions, and identity | 4 | 8 | 3 | 15 | *“I think it's good that […] is trying to keep the language and culture alive. I think that's really good.”* – Adult #2 |
| Environmental Awareness | Awareness and acknowledgement of environmental changes and their influences on the St. Paul Island food system | 4 | 6 | 5 | 15 | *“Less crab, less halibut, less seals. [...] It's the environment, the warmer oceans. I'm really glad the ice pack came. I'm really hoping it helps our ocean this year.”* – Adult #3 |
| Appreciation and Respect for Nature and the Environment | Appreciation, respect, and preservation of nature, wildlife, and the non-human environment | 2 | 5 | 4 | 11 | *“We have to respect our animals, our land and water.”* – Adult #7 |
| Work Ethic and Dedication | Commitment to wage labor and subsistence food procurement despite risks and challenges | 2 | 5 | 3 | 10 | *“I like helping around and doing stuff. […] Helping around just with anything, anybody needs, like bagging [at the community seal harvest] or any other thing.”* – Youth #4 |
| Authenticity in Food Sources | Preference of natural and traditional food sources | 2 | 3 | 3 | 8 | *“With subsistence, […] I like that a lot better [than grocery store foods] because I know what it is and what's put in it.”* – Youth #4 |
| **Instrumental Value** | **Definition** | **Youth (n=5)** | **Adults (n=9)** | **Elders (n=5)** | **Total Count** |  |
| Good Taste | Dietary choices and preferences for certain foods due to the satisfaction derived from the consumption of delicious foods | 4 | 7 | 5 | 16 | *“Oh my God. […] It's good, good food. I love my favorite foods. It tastes good.”* – Elder #1 |
| Community Support and Sharing | Receiving and sharing food and support among St. Paul Island community members for one's food security | 3 | 7 | 5 | 15 | *“Any subsistence foods were pretty much shared by the community with my family, with my dad. […] My dad got some great dishes of food from some wonderful cooks.”* – Elder #4 |
| Fun and Joy | Engagement in food system activities, including subsistence activities, and community food events, for fun and joy | 2 | 7 | 4 | 13 | *“I enjoy being on the water, so it's never work for me. You know, it's all joy.”* – Adult #6 |
| Autonomy and Independence | Self-sufficiency, autonomy, and independence in accessing local food sources for one's food security | 3 | 3 | 5 | 11 | *“I want to make sure that my girls know how to provide for themselves. That's what drives me to hunt. The main reason I do hunt now is just for survival.”* – Adult #4 |
| Environmental Sustainability | Sustainability and stability of nature, wildlife, and the non-human environment for the sustenance, survival, and security of the St. Paul Island community in current and future generations | 0 | 6 | 4 | 10 | *“And the ice coming down is good for the water temperatures and all the copepods and the small animals that feed the fish, birds, and stuff like that. So maybe we will see more birds surviving. And if we get more fish, then more seals will survive and stuff like that.”* – Elder #2 |
| Economic Sustainability | Sustainability and stability of the local economy for the sustenance, survival, and security of the St. Paul Island community in current and future generations | 0 | 7 | 3 | 10 | *“Halibut is very important to our community economy as well. And so is crab. […] But last year there was no halibut fisheries. […] And I heard there's no fishing season again this year. And if there's no fishing season, there's no halibut for us to eat.”* – Adult #3 |
| Health and Wellbeing | Dietary choices and preferences for certain foods due to their anticipated health benefits | 2 | 5 | 3 | 10 | “*What they have there keeps the very few of us, Unangan, that are living on this planet going in a healthy way, versus becoming diabetic, obese, having other health issues.”* – Elder #4 |
| Cultural Sustainability | Sustainability and stability of the traditional Aleut culture, practices, language, traditions, and identity for the sustenance, survival, and security of the St. Paul Island community in current and future generations | 3 | 3 | 3 | 9 | *“There are people here that are trying to bring it [Unangam tunuu] back, you know, and they're, you know, they're making a difference, I would say.”* – Adult #6 |
| Food Sovereignty and Security | Sovereignty over the local food system by the St. Paul Island community for the food security of current and future generations | 0 | 6 | 2 | 8 | *“I like to always think of that comparison of how our people lived and what they perceived and the honor they gave and using the entire animal. And that's food security.”* – Adult #7 |
| Advocacy for Change | Advocation for the awareness of and the involvement in addressing cultural and environmental issues for the sustenance, survival, and security of the St. Paul Island community in current and future generations | 0 | 4 | 3 | 7 | *“We keep going back to the meetings of, […] North Pacific Fishery Management Council and we talk about […] cutting back [bycatch]. So hopefully, you know, things will start working out.”* – Elder #2 |
| Innovation and Adaptability | Innovation and adaptation of the St. Paul Island community to environmental and societal challenges for the sustenance, survival, and security of the St. Paul Island community in current and future generations | 1 | 4 | 2 | 7 | *“Maybe someone should start farming, you know. […] That might be something to look into actually.”* – Adult #4 |
| Practical Engagement with Subsistence | Engagement in subsistence practices for practical reasons, including boredom, or to alleviate high food costs | 4 | 2 | 1 | 7 | *“It's just. I’m bored. Honestly. I like hunting. If I really wanted to connect to my spiritual roots, I'd build myself a spear and a kayak and go get a seal.”* – Youth #1 |
| Resilience | Resilience of the St. Paul Island community in the face of changes and disturbances for the sustenance, survival, and security of the St. Paul Island community in current and future generations | 0 | 3 | 1 | 4 | *“I would wish they would make it like the old days.”* – Elder #5 |
| Neutrality towards Food Choices | Indifference towards the types of food consumed | 1 | 0 | 0 | 1 | *“I don’t care what people eat.”* – Youth #5 |
| **Relational Value** | **Definition** | **Youth (n=5)** | **Adults (n=9)** | **Elders (n=5)** | **Total Count** |  |
| Community Cohesion, Collaboration and Support | Appreciation of community cohesion, collaboration, and support to maintain meaningful relations, often in adherence to traditional subsistence practices and sharing | 5 | 7 | 5 | 17 | *“It's not the taste. Kind of a taste. It has to do with the taste, but also just the experience I had with my family.”* – Youth #2 |
| Knowledge Transmission and Intergenerational Learning | Transmission and exchange of Indigenous, Traditional, and Local Knowledge (ILTK) among community members, including intergenerational learning opportunities | 4 | 8 | 5 | 17 | *“It's like, there's a certain feeling to it when I get to do this [sealing]. This tradition still lives on and I'm the next generation to teach it to my kids […] or my grandkids.”* – Youth #2 |
| Cultural Relations | Engagement with traditional Aleut culture, practices, language, and traditions to maintain one's identity, relations, and emotional and spiritual connections | 3 | 8 | 3 | 14 | *“Oh my gosh, it's [hunting] everything. It's us, our identity, how we grew up, how we subsisted off the land.”* – Elder #1 |
| Food Relations | Dietary choices and preferences for certain foods to maintain one's identity, relations, and emotional and spiritual connections | 2 | 7 | 4 | 13 | *“Mh, tastes like home. […] It's a type of soul food, you know, it's the food around here, even though it might not be healthy, people love it. It speaks to the heart.”* – Adult #5 |
| Community Responsibility and Provision | Support of St. Paul Island community members, including the provision and sharing of subsistence foods, due to a sense of responsibility | 2 | 6 | 3 | 11 | *“Since I have my own boat, it kind of feels like I have no choice but to provide.”* – Adult #6 |
| Environmental Relations | Engagement with nature, wildlife, and the non-human environment to maintain one's identity, relations, and emotional and spiritual connections | 1 | 4 | 3 | 8 | *“I just feel connected with nature when I do it [sealing].”* – Youth #2 |
| Leadership and Advocacy | Local Indigenous leadership and advocacy in community rights, societal challenges, cultural, and environmental issues through good relations, emotional, and spiritual connections | 0 | 2 | 4 | 6 | *“A great hope I have is that we can be leaders of a resistance. Of protecting our waters, like physically. Going out in boats or blockading, getting in the ways of these trawlers. I see that as a non-violent direct action. […] And so I feel like we as Indigenous people would need to lead that.”* – Adult #9 |
| Empowerment, Emancipation and Gender Equality | Awareness and appreciation of changing gender stereotypes, including the increasing role of women in subsistence activities, and its value for human-nature and human-human relations | 0 | 2 | 1 | 3 | *“I'm sure the women did the cooking and stuff, back then. But so when I started to partake in the harvest, […], I was really proud. Like, I'm a female. I could probably skin the seal faster than he could. […] And so I definitely carry that pride.“* – Adult #3 |

**Appendix G.** Quotes indicating changes in the St. Paul Island food system over time as identified from the stories and narrative interviews with youth, non-Elder adults, and Elders. Despite these changes in the local food system, the values listed in **Appendix F** persist.

*“It definitely changed our food system because introducing American foods, lactose, milk and stuff, which we did not have. And our bodies are still not used to it, even to this generation. It changed our way of life. We used to be, we used to live off the land and we used to like the land provided us, we provided the land. And now our land has just, we don't do that anymore. The food here is not even good. Prices are too high. It's hard to get stuff here. Fresh produce is horrible. Because of the Americans, because they colonized us. And it's just such a chain reaction where it just it's all falling.”* - Youth #2

*“It was very traditional […], the women stayed home and cooked and cleaned and they raised their daughters to the same exact thing. And now, […], like there's different roles. A lot of more women are working, a lot more women are doing things that men could do. You know, so in that aspect, […] like with how roles are nowadays and kids and people not really hunting anymore, it's not something that's really, really needed more so like to everyone's preference, you know? And so now we have like the ground beef at the store, we have the chicken at the store, you know, whereas before the majority of the community would live off the halibut and seal and whatever it was available at that season.”* - Non-Elder adult #1

*“I think our Aleut culture and language is just dying. Kids aren't really into traditional language and culture nowadays. They're just into internet and gaming, of course, and being on their phones, social media, it just takes it away from everyone. Yeah, internet and gaming and stuff like that. It's taking the kids away from learning their and being interested in their culture and everything. I think it's just the normal way of life now. Just so much technology now. It's just taking everyone away from their culture.”* - Non-Elder adult #2

*“Halibut is very important to our community, or our community economy as well. And so is crab. [It’s what] so many men in this community only worked for during the summer. […] And that's changed over more recent years because things have gotten more expensive. But last year, there was no halibut fisheries. […] and I heard, there's no fishing season again this year. Also, if there's no fishing season. There's no halibut for us to eat.”* - Non-Elder adult #3

*“We're getting less and less people every year who want to partake in the seal harvest. A couple of times they had to cancel because they couldn't get enough people who wanted to join. […] And if that continues then that might stop altogether, I mean, the harvest might stop. Certainly, some people might still go hunting for it, like with rifles and whatnot. But.”* - Non-Elder adult #5

*“There's not really much young commercial fishermen here anymore. It's kind of like a dying breed of fishing, like there's not many younger kids getting into it. […] It almost seems to be dying out, you know, here in St. Paul anyways.”* - Non-Elder adult #6

*“I guess if there is no commercial fishing […] people continue to keep moving. […] There's not going to really be much people that do subsistence anymore when it comes to fish and hunting. […] There's an older generation and then a few guys around my age that hunt and that's about it.”* - Non-Elder adult #6

*“People are living off the stuff they buy at the store. And, you know? Local food, teaching your kids how to cook it […] It's not getting done hardly anymore. So they kind of shy away from eating local stuff when they're too used to eating what they get out of the store.”* - Elder #5

*“Fewer and fewer people are wanting to go out and hunt and fish. Just people that commercially fish. Even some of them that do fish don't care too much about the fish they catch. They'll take it home and eat it. But they rather buy stuff from the store.”* - Elder #5

**References**

Abson DJ, Fischer J, Leventon J, Newig J, Schomerus T, Vilsmaier U, von Wehrden H, Abernethy P, Ives CD, Jager NW, Lang DJ (2017) Leverage points for sustainability transformation. Ambio 46: 30-39

Meadows, D (1999) Leverage Points: Places to Intervene in a System

Zimmermann S, Dermody B, Davis N, Divine LM, Padula VM, Lestenkof-Zacharof K, Wassen M, Dorresteijn I (2024) The importance of diversity in local food systems: A network analysis of food-related interactions in the Indigenous food system on St. Paul Island, Alaska. Reg Environ Change
